# A Ternary Hybrid EEG-NIRS Brain-Computer Interface for the Classification of Brain Activation Patterns during Mental Arithmetic, Motor Imagery, and Idle State

**DOI:** 10.3389/fninf.2018.00005

**Published:** 2018-02-23

**Authors:** Jaeyoung Shin, Jinuk Kwon, Chang-Hwan Im

**Affiliations:** Department of Biomedical Engineering, Hanyang University, Seoul, South Korea

**Keywords:** brain-computer interface, mental arithmetic, motor imagery, electroencephalography (EEG), near infrared spectroscopy (NIRS), pattern recognition

## Abstract

The performance of a brain-computer interface (BCI) can be enhanced by simultaneously using two or more modalities to record brain activity, which is generally referred to as a hybrid BCI. To date, many BCI researchers have tried to implement a hybrid BCI system by combining electroencephalography (EEG) and functional near-infrared spectroscopy (NIRS) to improve the overall accuracy of binary classification. However, since hybrid EEG-NIRS BCI, which will be denoted by hBCI in this paper, has not been applied to ternary classification problems, paradigms and classification strategies appropriate for ternary classification using hBCI are not well investigated. Here we propose the use of an hBCI for the classification of three brain activation patterns elicited by mental arithmetic, motor imagery, and idle state, with the aim to elevate the information transfer rate (ITR) of hBCI by increasing the number of classes while minimizing the loss of accuracy. EEG electrodes were placed over the prefrontal cortex and the central cortex, and NIRS optodes were placed only on the forehead. The ternary classification problem was decomposed into three binary classification problems using the “one-versus-one” (OVO) classification strategy to apply the filter-bank common spatial patterns filter to EEG data. A 10 × 10-fold cross validation was performed using shrinkage linear discriminant analysis (sLDA) to evaluate the average classification accuracies for EEG-BCI, NIRS-BCI, and hBCI when the meta-classification method was adopted to enhance classification accuracy. The ternary classification accuracies for EEG-BCI, NIRS-BCI, and hBCI were 76.1 ± 12.8, 64.1 ± 9.7, and 82.2 ± 10.2%, respectively. The classification accuracy of the proposed hBCI was thus significantly higher than those of the other BCIs (*p* < 0.005). The average ITR for the proposed hBCI was calculated to be 4.70 ± 1.92 bits/minute, which was 34.3% higher than that reported for a previous binary hBCI study.

## Introduction

Brain-computer interfaces (BCIs) have recently attracted great attention as they have shown great potential as new modes of communication for individuals who have lost the ability for voluntary movements (Wolpaw et al., 2002; Allison et al., 2007; van Erp et al., 2012; Wolpaw and Wolpaw, 2012; Blankertz et al., 2016). BCIs can be implemented using a variety of neural signal recording methods, such as electroencephalography (EEG), magnetoencephalography, functional magnetic resonance imaging, near-infrared spectroscopy (NIRS), electrocorticography, and multiunit neural recording (Wolpaw et al., 2002). Since each brain-imaging modality has its own pros and cons, combining two or more neural signal recording modalities, which is generally referred to as hybrid BCI, might enhance the overall performance of BCI (Pfurtscheller et al., 2010a; Dähne et al., 2015; Fazli et al., 2015). Until now, a number of hybrid BCI studies have demonstrated the effectiveness of the combinatory use of different modalities or paradigms, e.g., combined use of P300 and steady-state visually evoked potential for EEG-BCI (Wang et al., 2015), hybrid EEG-electrooculogram (EOG) BCI (Wang et al., 2014), and hybrid EEG-NIRS BCI (hereafter denoted by hBCI) (Naseer and Hong, 2015).

Among the different methods, hBCI has been actively studied because both modalities can be readily made portable and there is no significant interference between the two signals. EEG records electrophysiological signal and NIRS measures hemodynamic variations in the brain. Since the origins of the two signals differ from each other, the amount of available information that can be used for BCI is increased, which in turn leads to enhanced BCI performance. More importantly, the two modalities are complementary to each other in that EEG has superior temporal resolution to NIRS, but is more prone to contamination from EOG and electromyogram artifacts than NIRS. Indeed, recent studies reported successful application of NIRS-BCI for the communication of patients in completely locked-in state (CLIS) (Chaudhary et al., 2017); however, EEG-BCI has never been successful for the patients in CLIS (De Massari et al., 2013; Chaudhary et al., 2017). Therefore, the appropriate combination of these two modalities has the potential to enhance the overall BCI performance and it has been already verified in many previous studies (Fazli et al., 2012; Koo et al., 2015; Yin et al., 2015; Shin et al., 2016, 2017b). The recent release of an open-access dataset for hBCI reflects the increasing attention that this type of hBCI has garnered (Shin et al., 2017a).

The easiest way to increase the information transfer rate (ITR) of a BCI system is to increase the number of classes, while minimizing the loss of accuracy, because ITR is determined by both classification accuracy and the number of available commands; however, most hBCI studies have focused only on enhancing the classification performance of binary BCI. Until now, hBCI has been studied to improve the classification performance of binary BCI (Shin et al., 2017b) or to simply increase the number of available commands (Khan et al., 2014; Khan and Hong, 2017). Khan et al. (2014, 2015) recorded EEG and NIRS simultaneously but they used the two types of data independently. Here we propose the use of a multi-class hBCI that classifies three brain activation patterns recorded during motor imagery (MI), mental arithmetic (MA), and idle state (IS: staying relaxed without performing any cognitive task). MI has been the most widely used mental task for EEG-BCI, while MA has been frequently used as a popular BCI task for NIRS-BCI (Power et al., 2011, 2012a,b). Brain activation elicited by MI can be measured mainly around the central area, while that elicited by MA can be measured primarily in the forehead covering the prefrontal cortex (PFC). Therefore, we hypothesized that ternary classification (MA vs. MI. vs. IS) would be suitable for implementation of ternary hBCI. In contrast to EEG, which can record brain activity from both the frontal and central areas relatively easily, NIRS sometimes has difficulty in measuring signals around the central areas due to the attenuation of light intensity by hair, without applying a time-consuming hair preparation process or using specially designed brush type optodes (Khan et al., 2012). In this study, we implement an hBCI using EEG signals recorded at both the frontal and central areas and NIRS signals recorded only from the frontal area to improve the practicality and usability of the system by avoiding time-consuming (hair) preparatory work. The performance of our hBCI was validated using experiments with 18 healthy participants.

## Materials and methods

### Participants

Eighteen healthy participants (10 men and 8 women, 23.8 ± 2.5 years of age) voluntarily participated in this study. None of the participants reported a history of neurological, psychiatric, or other severe diseases that might have influenced the experimental results. The experimental procedure was fully explained to each participant before the experiment. The participants signed written consent forms before the experiment. After the experiment, monetary reimbursement was provided. The experiment was conducted with approval from the Institutional Review Board committee of Hanyang University and according to the Declaration of Helsinki.

### Apparatus

**Figure 1:**
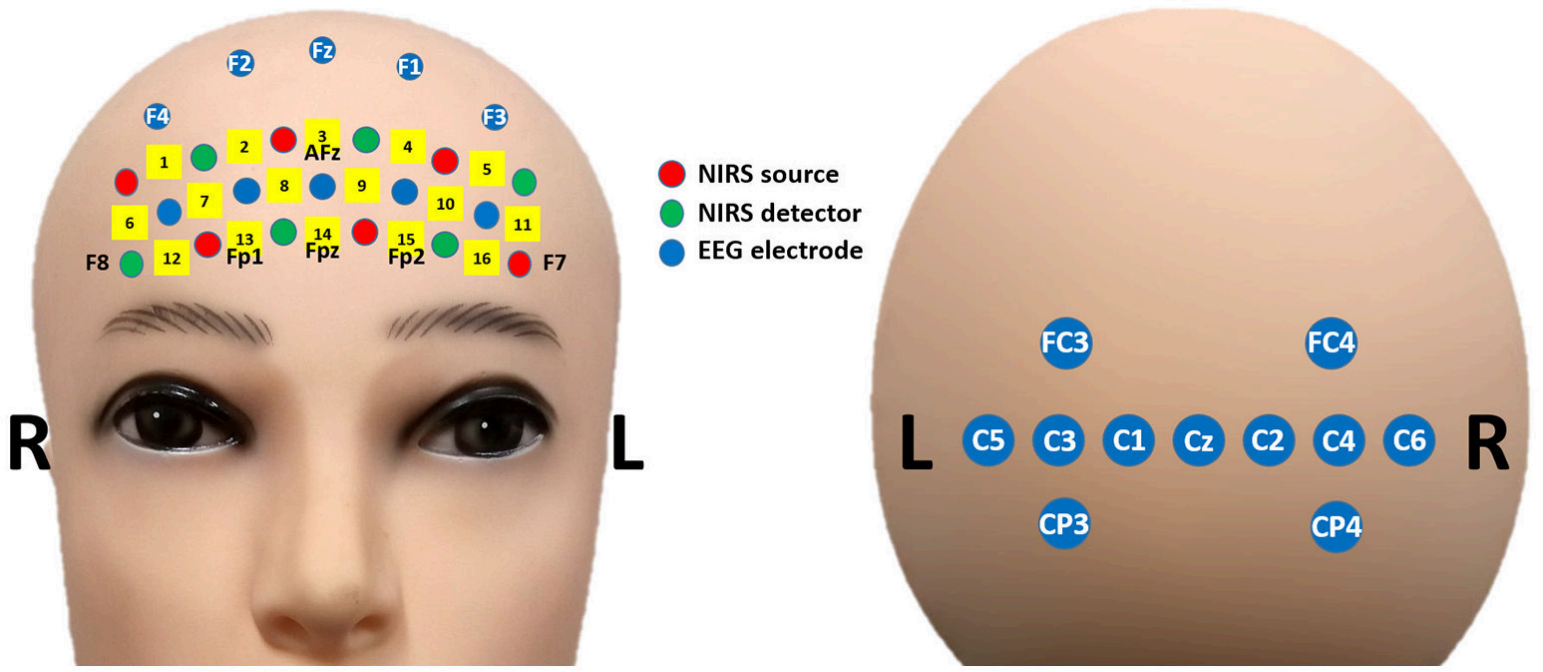
Placement of EEG electrodes (blue) and NIRS optodes (red: sources, green: detectors) on frontal **(Left)** and central **(Right)** areas.

Figure 1 shows the placement of the EEG electrodes and NIRS optodes. EEG data were recorded at a sampling rate of 2,048 Hz using an Active-Two amplifier (Biosemi; Amsterdam, the Netherlands) with 21 active electrodes placed on both frontal [5 unlabeled (non-standard), Fz, F1, F2, F3, and F4] and central (FC3, FC4, Cz, C1, C2, C3, C4, C5, C6, CP3, and CP4) areas. The reference and ground electrodes were attached at the left and right mastoids, respectively. Two additional electrodes were located above and below the left eye to measure the vertical EOG. NIRS data were collected using a portable NIRS system (LIGHTNIRS; Shimadzu Corp.; Kyoto, Japan) at a sampling rate of 13.3 Hz. Six sources and six detectors were placed on the forehead over the PFC. There were 16 NIRS channels in total. Each of these channels consisted of a source and detector pair placed 30 mm away from each other. To synchronize the two signals, trigger signals were delivered to both the EEG and NIRS systems simultaneously using StimTracker (Cedrus Corp.; San Pedro, USA).

### Experimental paradigm

**Figure 2:**
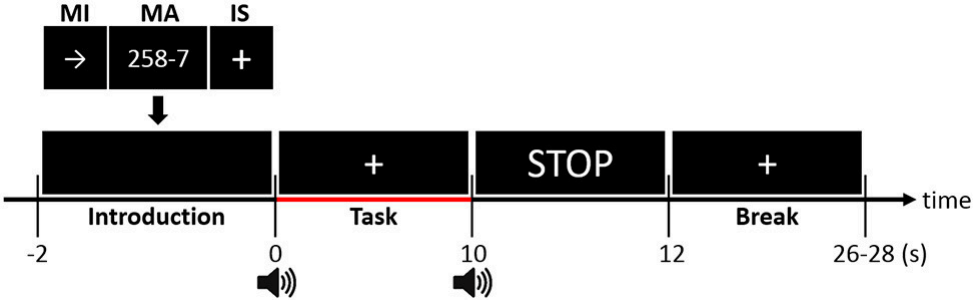
Timing sequence of a single trial. A random task was assigned to the participant in the Introduction section (−2 to 0 s). After the presentation of a short beep, the participants continued performing the assigned task while looking at a fixation cross during the task period (0–10 s). The participants stopped performing the task after a second short beep was presented and a “STOP” sign was displayed on the screen for 2 s. During the random-length inter-trial break period (12 to 26–28 s), the participants relaxed without any particular thoughts.

The participants were seated on a comfortable chair 70 cm away from a 26-inch liquid crystal display monitor and followed instructions appearing on the monitor. Figure 2 shows the experimental paradigm. A single trial was composed of instruction (−2 to 0 s), task (0–10 s), and inter-trial break (10 to 26–28 s) periods. In the introduction period, a right-hand MI, MA, or IS was randomly selected. For the right-hand MI, a right arrow was presented, and for the MA, “a three-digit number minus a one-digit number (between 6 and 9)” was randomly provided. For IS, a fixation cross was displayed at the center of the monitor. In the task period, the participants performed the designated task. For the right-hand MI, the participants imagined complex finger tapping (tapping the second, third, fourth, fifth, fourth, third, second, etc. fingers to the thumb) at a rate of approximately 2 Hz. For MA, the participants were instructed to continuously subtract a one-digit number (between 6 and 9) from the result of a former calculation as fast as possible (e.g., 789–7 = 782, 782–7 = 775, 775–7 = 768). For IS, the participants stayed relaxed without performing any specific mental imagery task. The participants performed the three types of tasks 30 times each (90 times in total). Note that a number of NIRS-BCI and hBCI studies have adopted IS as one of the main BCI tasks (Power et al., 2011; Schudlo et al., 2013; Schudlo and Chau, 2015). Before the experiment, all participants were pre-trained to produce appropriate MI-related brain activation patterns with the aid of a visual feedback system. For the visual-feedback-based MI training, three EEG electrodes (Cz, C3, and C4) were selected to monitor the motor-related EEG signal changes. At first, task-related event-related synchronization/desynchronization (ERS/ERD) changes at the three electrodes were displayed on the monitor as simple bar graphs, which were updated in real-time while participants performed actual finger tapping. Once the participants got used to the task, they performed kinesthetic MI, not visual MI (Neuper et al., 2005), to make ERS/ERD patterns similar to those generated during actual finger tapping. If they could reproduce consistent task-related ERS/ERD patterns, data recording commenced. The effectiveness of the MI training (MI proficiency) was evaluated based on the elapsed training time (good: < 5 min, normal: < 20 min, poor: < 30 min). The total training time was limited to at most 30 min considering participants' attentional deterioration and fatigue, but most participants of this study finished the MI training session within 20 min (normal MI proficiency).

### Preprocessing

All data processing was performed using MATLAB, 2013b (MathWorks; Natick, MA). Functions implemented in EEGLAB (https://sccn.ucsd.edu/eeglab/index.php) and BBCI^1^ toolbox (https://github.com/bbci/bbci_public) were used for EEG and NIRS data processing and classification (Delorme and Makeig, 2004; Blankertz et al., 2016). EEG data were downsampled to 200 Hz to reduce the computational complexity and band-pass filtered with a passband of 0.1–50 Hz to remove direct current drift and 60 Hz alternating current noise. The vertical EOG was eliminated using an automatic ocular artifact rejection method based on a blind source separation algorithm (Gomez-Herrero et al., 2006). For NIRS, the detected optical densities (ODs) were converted to hemodynamic variations (concentration change in reduced hemoglobin ΔHbR and concentration change in oxidized hemoglobin ΔHbO) using the following formula (Matcher et al., 1995):

$$\begin{array}{l} \left(\begin{array}{c} \Delta \text{HbR}\\ \Delta \text{HbO} \end{array}\right)=\left(\begin{array}{ccc} 1.8545 & -0.2394 & -1.0947\\ -1.4887 & 0.5970 & 1.4847 \end{array}\right)\\ \;\left(\begin{array}{c} \Delta \text{O}{\text{D}}_{780}\\ \Delta \text{O}{\text{D}}_{805}\\ \Delta \text{O}{\text{D}}_{830} \end{array}\right)\left(\text{mM }\cdot \text{ cm}\right) \end{array}$$

In the equation above, ΔOD is the change in the detected OD at the wavelength provided in the subscript (780, 805, or 830 nm). The converted ΔHbR and ΔHbO values were band-pass filtered (6th-order Butterworth zero-phase filter) with a passband of 0.01–0.09 Hz to remove physiological noise.

### Classification

**Figure 3:**
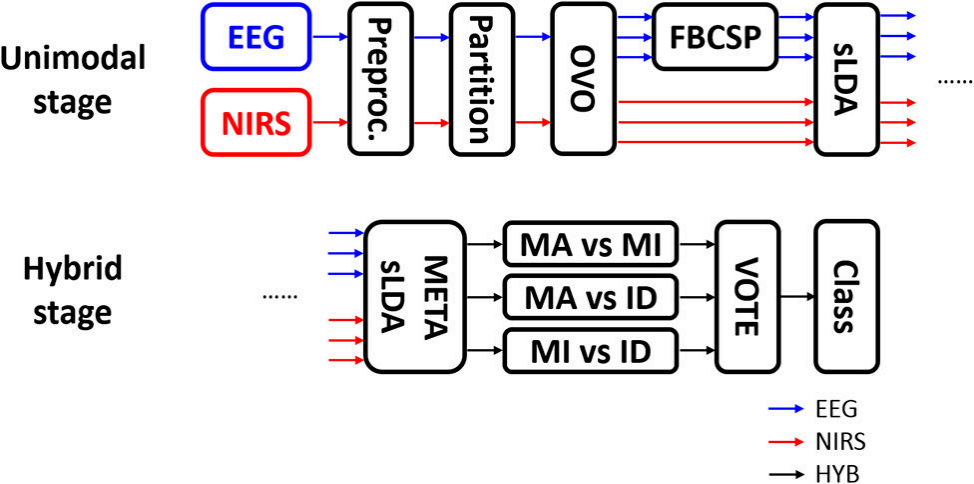
Data processing flow. EEG and NIRS data were separately processed at the unimodal stage and were combined at the hybrid stage. OVO, FBCSP, and sLDA indicate “one-versus-one” strategy, filter-bank common spatial pattern, and shrinkage linear discriminant analysis, respectively. After OVO, the ternary classification problem was decomposed into three binary classification problems.

Figure 3 shows the procedure used for data processing and classification. EEG data were segmented into epochs from −5 to 25 s. To apply the filter-bank common spatial pattern (FBCSP) filter, EEG data in the time period 0–10 s (i.e., task period) were selected and a filter bank (6th-order zero-phase Butterworth) with multiple passbands for the θ (4–8 Hz), α (8–13 Hz), and β (13–30 Hz) bands was applied to the selected EEG data. EEG epochs were partitioned into training and test sets. The ternary classification problem was decomposed into three binary classification problems (i.e., MA vs. MI, MA vs. IS, and MI vs. IS) in order to apply the “one-versus-one” (OVO) classification strategy (Müller-Gerking et al., 1999; Dornhege et al., 2004), in which the classification was performed for all possible binary combinations of classes and the final estimate was decided by majority voting (Lei et al., 2009). EEG feature vectors {dimension: 18 × 60 [(number of CSP components × number of passbands) × number of trials]} were constructed using the log-variance of the first three and last three CSP components selected using the typical eigenvalue score (Blankertz et al., 2008) after FBCSP filtering.

NIRS data were segmented into epochs from −5 to 25 s. Baseline correction was performed by subtracting the temporal mean value between −1 and 0 s from each NIRS epoch. NIRS feature vectors {dimension: 64 × 60 [(number of channels × number of NIRS chromophores × number of temporal windows) × number of trials]} were constructed using the temporal mean values of HbR and HbO in the 5–10 and 10–15 s temporal windows in NIRS epochs from all channels, considering the inherent hemodynamic delay. Note that we also tried other feature candidates such as slope and variance but the use of features other than the temporal mean values did not improve the classification performance. In addition, the two separated intervals (5–10 and 10–15 s) yielded higher classification accuracy than a single interval (5–15 s). In the same manner as that described for EEG data above, NIRS epochs were partitioned to training and test sets, and the OVO classification strategy was applied. We adopted the OVO approach because the OVO strategy makes CSP, known to yield high performance in ERS/ERD-based BCI, be applied to the present ternary classification problem.

A 10 × 10-fold cross-validation was performed using shrinkage linear discriminant analysis (sLDA) for each of the three binary classification problems. The sLDA can be used to effectively mitigate the negative effect (degradation of classification accuracy) resulting from the use of high-dimensional feature vectors when compared to the number of trials by replacing the empirical covariance matrix Σ with (1−λ)Σ+λ*I*, where λ and *I* are the regularization parameter and identity matrix, respectively (Friedman, 1989; Shin et al., 2016, 2017a,b). The optimal λ was estimated based on the literature (Ledoit and Wolf, 2004; Schäfer and Strimmer, 2005). sLDA classifiers were trained by EEG and NIRS data separately for three binary classification problems each, and the outputs of EEG and NIRS classifiers were then combined to construct new feature vectors for the meta-classifier (Fazli et al., 2012). The final class was then estimated using majority voting for the results of the three binary classification problems.

## Results

**Figure 4:**
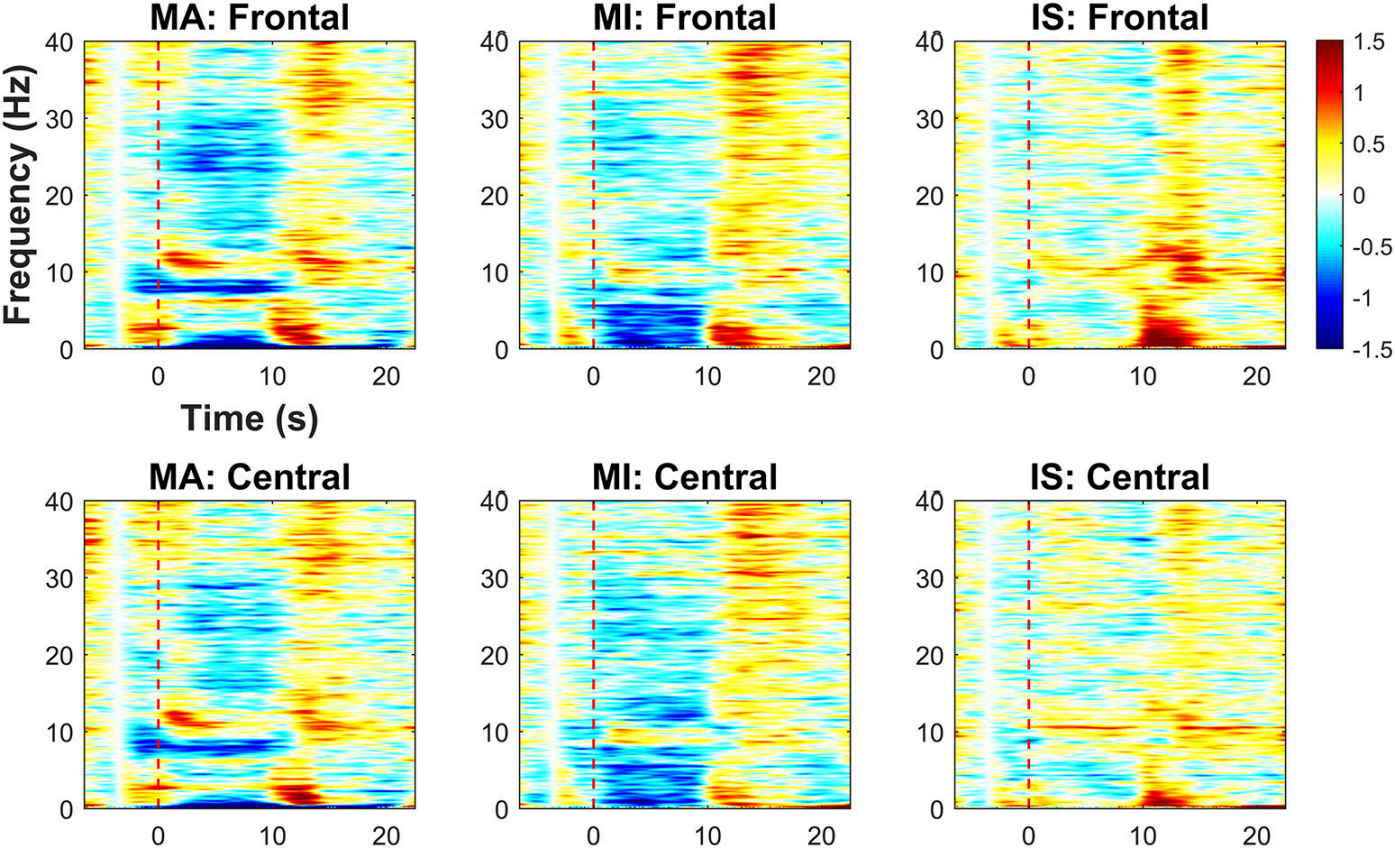
Time-frequency analysis results for MA, MI, and IS on frontal and central areas (unit: dB). Red dashed lines represent the task onset. A color bar indicates the range of the EEG spectral power variation in dB. Red (positive) and blue (negative) indicate, respectively, spectral power increases and decreases relative to the baseline value (average spectral power between −4 and −3 s).

Figure 4 shows the results of the time-frequency analysis, which is used to assess task-related EEG spectral power changes over the frontal and central areas relative to the baseline value, which is the average spectral power between −4 and −3 s. Red and blue colors indicate increases and decreases in EEG spectral power, respectively. The spectral power changes were averaged over the frontal and central channels separately. A red dotted vertical line in each graph indicates the task onset time (0 s). For MA, power decreases in the δ- (1–4 Hz) and low α-bands (8–10 Hz) were observed over frontal and central areas during the task period. Power increases in the high α-band (10–13 Hz) were commonly seen in the early stages of the task period (0–5 s). Power decrease in the high β-band (20–30 Hz) appeared more distinctly in the frontal area than in the central area. For MI, prominent power decreases in the δ- and θ-bands were observed over both frontal and central areas during the task period. Similar spectral power changes in the frontal area during MI was observed in previous studies (Yamawaki et al., 2006; Ahn et al., 2013). In addition, a power decrease in the high α-band was observed in the central area. For IS, no distinct power change was observed during the task period, except for a weak power increase around 10 Hz, which might have been due to the relaxation of the participants (Lagopoulos et al., 2009). Regardless of the task type, power increases commonly appeared throughout the frontal and central areas after the end of the task period. The power increase in the δ-band might be the remaining (not completely eliminated by ICA) EOG components due to eye blinking right after the task period. Note that this δ-band power increase did not affect the BCI performance because frequency bands higher than θ-band were used for the BCI classification. The ERS in the α frequency band seems to be the post-stimulus ERS frequently observed after performing a given task (Pfurtscheller et al., 2006; Solis-Escalante et al., 2010; Shin et al., 2017b).

**Figure 5:**
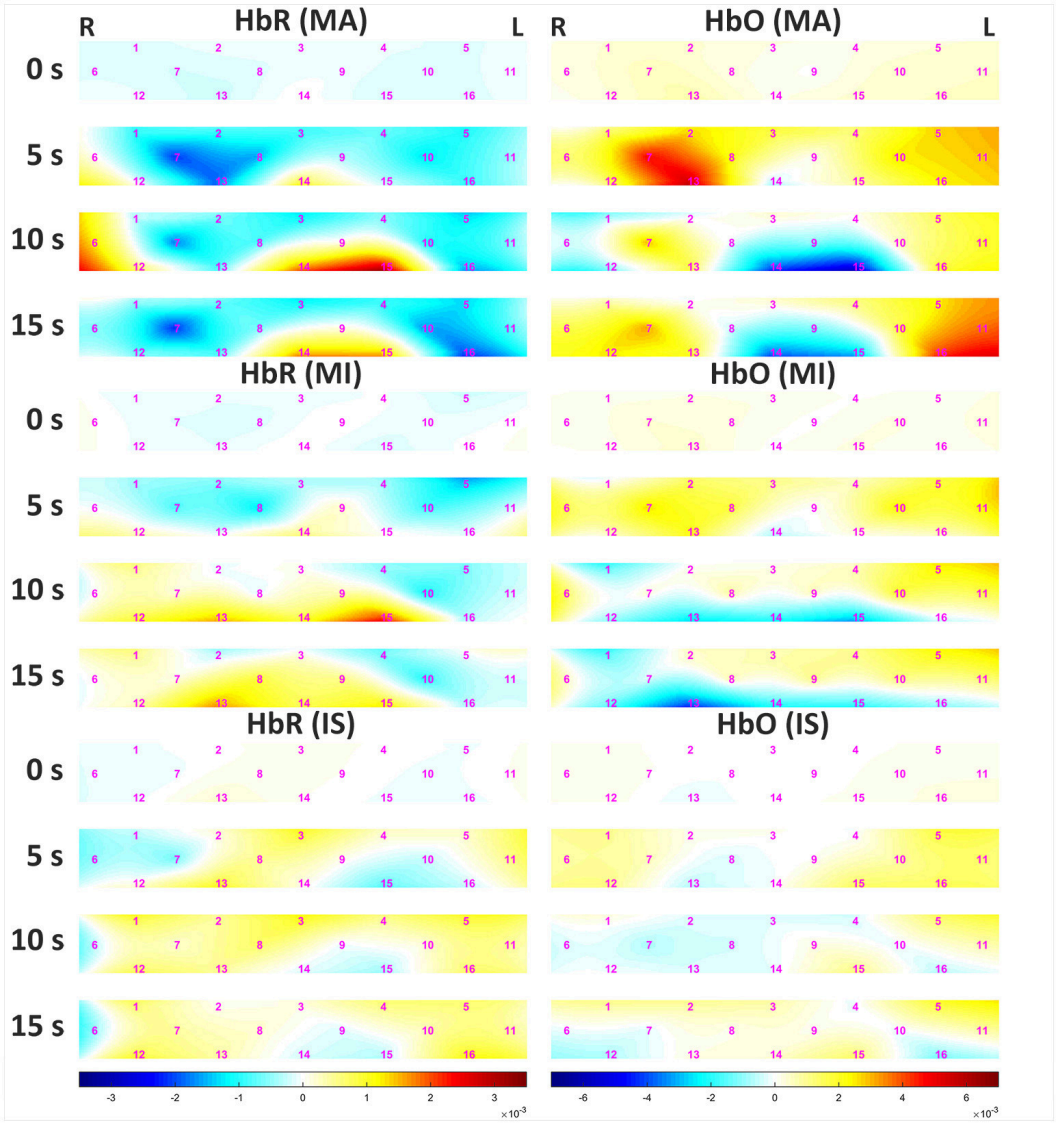
Grand average of hemodynamic variation in response to MA **(Top)**, MI **(Middle)**, and IS **(Bottom)** over time. The left and right panels present data for HbR and HbO, respectively. The color bars below the figures indicate the range of the concentrations of HbR (left 3 panels) and HbO (right 3 panels) in mM·cm. Note that the ranges of the concentrations are different.

Figure 5 shows the grand average of hemodynamic variations due to MA and MI over time. The left and right panels represent data for HbR and HbO, respectively. Channels (Chs.) 3 and 14 are located in the middle of the forehead. For MA, the decrease in HbR appeared at center-right channels (Chs. 7, 8, and 13) 5 s after task onset. After 10 s, an increase in HbR was observed at the center-left-lower (Chs. 14 and 15) and rightmost (Ch. 6) channels. At 15 s, the amount of increase in HbR was reduced. For MI, minor variations in HbR were observed until 5 s after task onset. After 10 s, an increase in HbR was observed in the left lower channel (Ch. 15), but the variation was not prominent when compared to that observed for MA. At 15 s, the amount of increase in HbR was reduced. For both MA and MI, the trend of HbO variation was opposite to that observed for HbR, and the variation of HbO was greater than that of HbR. After 10 s, a sudden drop of HbO was observed on the left-middle area (chs. 9, 14, 15), which might seem unusual; however, note that some previous studies also reported similar phenomena during mental arithmetic task (Pfurtscheller et al., 2010b; Shin et al., 2017a). The grand averaged HbR and HbO waveforms at three different task conditions can be found at https://doi.org/10.6084/m9.figshare.5844813.v1.

**Figure 6:**
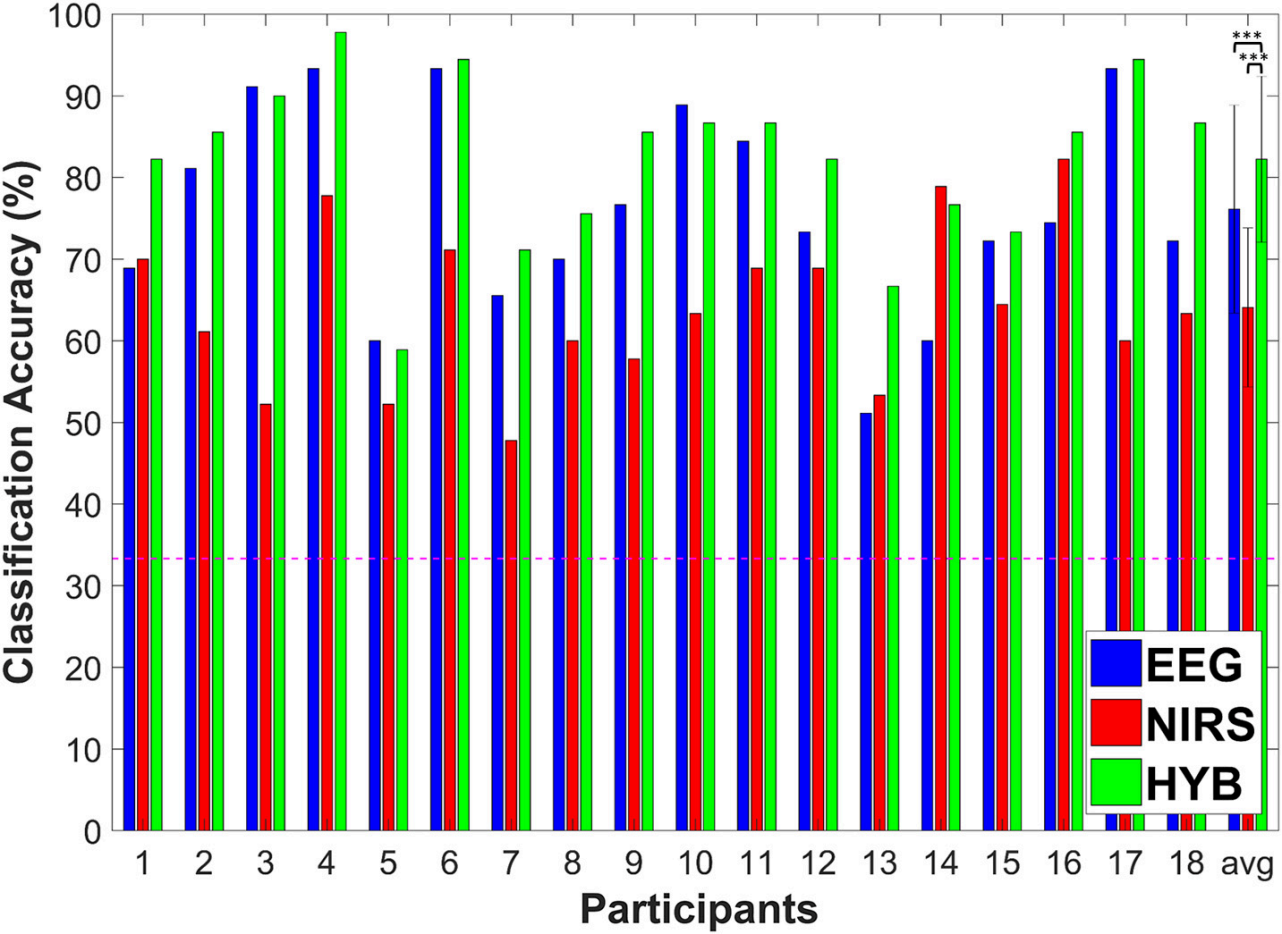
Individuation classification accuracy of EEG (blue), NIRS (red), and HYB (green), and the average for all participants. The horizontal dashed line indicates the theoretical chance level (33.3%). Errorbars indicate the standard deviation. ^***^p < 0.005.

Figure 6 shows the individual ternary classification accuracies for EEG-BCI, NIRS-BCI, and hBCI (denoted by HYB in Figure 6). A red dotted horizontal line denotes the theoretical chance level (1/3 = 0.333). As shown in the graph, we were able to use the “HYB” to obtain the best classification accuracies for 14 out of the 18 participants. The average classification accuracies for EEG, NIRS, and “HYB” were 76.1 ± 12.7, 64.1 ± 9.7, and 82.2 ± 10.2% (mean ± standard deviation), respectively. The average classification accuracy of hBCI was statistically significantly higher than those of EEG-BCI and NIRS-BCI (Friedman test: *p* < 0.001; *post-hoc*: Wilcoxon signed rank sum test with Bonferroni correction; EEG vs. HYB: corrected *p* = 0.0046 and NIRS vs. HYB: corrected *p* = 0.0002).

## Discussion

The results of our study show, for the first time, the feasibility of the combined use of EEG and NIRS to enhance the performance of ternary BCI classification. The proposed ternary hBCI was used to classify MA-, MI-, and IS-related brain activation patterns successfully with higher classification accuracy than EEG-BCI and NIRS-BCI. The MA-related brain activation and the MI-related activation are mainly produced in different brain regions, and thereby NIRS optodes and EEG electrodes were arranged on the forehead over the PFC to record MA-related brain activation, while additional EEG electrodes were placed on the central area to record MI-related brain activation. It is noteworthy that the ternary classification accuracy was even higher than the threshold for effective binary BCI (>70% classification accuracy) (Blankertz et al., 2009; Vidaurre and Blankertz, 2010).

The implementation of a multi-class NIRS-BCI is challenging. No previous study has successfully implemented a multi-class NIRS-BCI using only hemodynamic variations recorded from the PFC. The accuracy of our ternary classification using NIRS supports this argument. Although the average ternary NIRS classification accuracy exceeded the theoretical chance level (33.3%), a practically usable level was unreachable. To implement ternary NIRS-BCI, Hong et al. (2015) and Schudlo and Chau (2015) placed more NIRS optodes on central areas and the parietal cortex, respectively, in addition to the PFC. However, time-consuming preparation is generally inevitable to acquire high-quality signals if optodes are attached onto hairy scalp areas. On the other hand, although EEG-BCI resulted in a fairly high classification accuracy exceeding 70%, the classification accuracies for 9 out of the 18 participants were further improved by more than 5% using the hybrid approach.

Successful implementation of a multi-class hBCI is beneficial for improving the ITR given by 60/τ·[log_2_*N*+*p*log_2_(*p*) + (1−*p*)log_2_((1−*p*)/(*N*−1))] where τ, *N, p* and are trial length, the number of classes, and classification accuracy, respectively (Dornhege et al., 2007). According to a previous study (Hong et al., 2015), an average ITR of 3.29 ± 0.72 bits/min could be achieved using the ternary NIRS-BCI. The average ITR of 3.50 ± 1.23 bits/min was achieved using the binary hBCI based on MA (Shin et al., 2017a). We achieved an average ITR of 4.70 ± 1.92 bits/min, which reflects 42.9 and 34.3% improvements in ITR when compared to a ternary NIRS-BCI (Hong et al., 2015) and a binary hBCI (Shin et al., 2017a), respectively. Multi-class classification may generally degrade classification accuracy when compared to binary classification, although it can increase the number of available commands. In this study, we demonstrated that hBCI can mitigate the degradation of classification accuracy, thereby further improving the ITR. Although it might be thought that some EEG-BCI paradigms such as steady-state visual evoked potential (SSVEP)-BCI and P300 BCI can provide higher ITR (Ming et al., 2002; Hwang et al., 2012), they rely on exogenous paradigms requiring external visual stimulus (Faller et al., 2010). Therefore, they cannot be used for patients with oculomotor impairment or those in CLIS.

We selected MI and MA as two mental tasks producing distinct response, as they have been widely used in previous EEG-BCI or NIRS-BCI studies (Zhang et al., 2017; Dutta et al., 2018). However, in previous BCI studies, many other types of mental tasks (e.g., word association, mental singing, three-dimensional object rotation, mental navigation, and face imagery) were considered in order to find an optimal combination of BCI tasks (Friedrich et al., 2012; Hwang et al., 2014; Banville et al., 2017). It is expected that larger ITRs would be achieved if the optimal task combination and the optimal task length are established.

The main drawback of the current hBCI is the high complexity of the system, which might make it difficult to apply in practical BCI applications. To reduce the system complexity, we attached EEG electrodes only around frontal and central areas based on a previous hBCI study that used the same mental tasks as the present study (Shin et al., 2017a). Therefore, it is necessary to minimize the system complexity using an optimal channel selection method and by manufacturing a unified EEG-NIRS recording system. No commercial hybrid recording system able to collect EEG and NIRS data simultaneously in a single unit is available (von Lühmann et al., 2017). Overcoming this drawback would enable advancements in hBCI research in the future.

## Author contributions

This study was designed by JS and C-HI. The preliminary and main experiments and data analyses were conducted by JS and JK. The manuscript was written by JS and C-HI. All of the authors have reviewed and approved the final manuscript.

### Conflict of interest statement

The authors declare that the research was conducted in the absence of any commercial or financial relationships that could be construed as a potential conflict of interest.

## Footnotes

^1^BBCI toolbox [Online]. Available: https://github.com/bbci/bbci_public/
